# Implementing the Design of Experiments (DoE) Concept into the Development of Mucoadhesive Tablets Containing Orange Peel Extract as a Potential Concept for the Treatment of Oral Infections

**DOI:** 10.3390/ma17215234

**Published:** 2024-10-28

**Authors:** Magdalena Paczkowska-Walendowska, Tomasz M. Karpiński, Ewa Garbiec, Michał Walendowski, Judyta Cielecka-Piontek

**Affiliations:** 1 Department of Pharmacognosy and Biomaterials, Poznan University of Medical Sciences, Rokietnicka 3, 60-806 Poznan, Poland; egarbiec@ump.edu.pl (E.G.); jpiontek@ump.edu.pl (J.C.-P.); 2 Department of Medical Microbiology, Medical Faculty, Poznan University of Medical Sciences, Rokietnicka 10, 60-806 Poznan, Poland; tkarpin@ump.edu.pl; 3 Science-Bridge Sp. z o.o., Chociszewskiego 24/8, 60-258 Poznan, Poland; michalwalendowskipoczta@gmail.com

**Keywords:** buccal tablets, chitosan, orange peel extract, hydroxypropyl methylcellulose, dissolution, mucoadhesion, oral cavity infections, statistical design of experiments

## Abstract

This study explores for the first time the impact of chitosan (CS) with varying molecular weights (MW), orange peel extract concentration, and hydroxypropyl methylcellulose (HPMC) content on the formulation of buccal tablets for treating oral infections. Utilizing a statistical design of experiments (DoE), nine different formulations were evaluated for mechanical properties, dissolution behavior, mucoadhesion, and biological activity. A formulation with high CS MW, 60% orange peel extract, and 8% HPMC, emerged as the optimal formulation, demonstrating superior tabletability, compressibility, and compactibility. Dissolution studies indicated that hesperidin release followed the Higuchi model, with higher extract content enhancing this phenomenon. Mucoadhesion improved with increased HPMC and CS concentrations, although higher extract content reduced bioadhesion. Biological assays showed that higher extract levels boosted antioxidant activity, while CS primarily contributed to anti-inflammatory effects. The optimized formulation exhibited broad antimicrobial activity against key oral pathogens, surpassing the effectiveness of the individual components. Principal component analysis (PCA) further confirmed the significant influence of extract content on tablet properties. These findings suggest that the optimized tablet formulation holds promise for effective buccal delivery in the treatment of oral infections, warranting further investigation in clinical settings.

## 1. Introduction

Oral cavity infections, including periodontal disease, dental caries, and oral candidiasis, are prevalent health concerns that significantly impact quality of life [1]. It is estimated that oral diseases affect nearly 3.5 billion people [2]. Conventional treatments often involve systemic antibiotics or antiseptics, which can lead to undesirable side effects, including antibiotic resistance and disruption of the natural oral microbiota [3]. As a result, there is a growing interest in developing targeted, locally acting therapies that can deliver active ingredients directly to the site of infection while minimizing systemic exposure [4].

One promising approach is the use of buccal tablets, which adhere to the mucosal surface of the mouth and release therapeutic agents over an extended period [5]. These formulations offer several advantages, including prolonged contact with the affected area, enhanced bioavailability of the drug, and reduced dosing frequency [6]. Key to the success of buccal tablets is the careful selection and optimization of excipients that influence their mechanical properties, drug release profiles, and mucoadhesive characteristics [7]. Xanthan gum-based tablets rich in resveratrol offered a delayed and controlled release of the substance, together with favorable mucoadhesive properties [5]. While CS/HPMC-based tablets containing *Scutellariae baicalensis radix* extract provided a valuable baicalin release profile, and had good mucoadhesive qualities that condition the tablet’s retention in the application site and therapy efficacy [8]. Chitosan (CS), a natural biopolymer, has been widely recognized for its bioadhesive properties and ability to enhance drug permeation across mucosal tissues [9]. Previous studies have shown that the molecular weight of CS significantly affects its biological activities and mechanical properties [10]. It also has its own antimicrobial effect and is used in various indications to enhance this effect [11]. Additionally, hydroxypropyl methylcellulose (HPMC) is commonly used in pharmaceutical formulations to modulate drug release and improve mucoadhesion [12].

In light of the development of bacterial resistance to antibiotics, more and more attention is being focused on the use of plant materials, due to their complex biological activity, often favorable safety profile, lower therapy costs, biocompatibility, and low impact on the environment [13]. One of such plant materials is orange peel. Orange peel, considered as waste [14], but rich in bioactive compounds such as hesperidin, has demonstrated potent antioxidant, anti-inflammatory, and antimicrobial properties [15]. These attributes make it a potential candidate for incorporation into buccal tablets aimed at treating oral infections. However, the formulation of such tablets poses challenges, particularly in balancing their release kinetics and ensuring adequate mucoadhesion for sustained drug delivery [16].

In this study, it was hypothesized that buccal tablets containing orange peel extract can be successfully formulated by optimizing chitosan molecular weight, the ratio of orange peel extract to CS, and HPMC content. Specifically, it was expected that these factors will have significant effects on the tablets’ mechanical properties, dissolution behavior, and biological activity. By systematically evaluating these variables using a design of experiments (DoE) approach, the identification of an optimal formulation that maximizes therapeutic efficacy for oral cavity infections while ensuring feasibility in tablet production was anticipated.

## 2. Materials and Methods

### 2.1. Plant Material

*Aurantii amari* pericarpium et mesocarpium (orange peel) was purchased from Natura Wita, Zdrowie z Zielnika (Pinczow, Poland) (lot no. 270821).

### 2.2. Chemicals and Reagents

All chemicals, like excipients, such as chitosans (low molecular weight = LMW 20–300 cps, 1% in 1% acetic acid; medium molecular weight = MMW 200–800 cps; high molecular weight = HMW 800–2000 cps) and hydroxypropyl methylcellulose (average Mn~90,000, ~15,000 cP), and magnesium stearate, as well as reagents for activity assays (2, 2-Diphenyl-1-picrylhydrazyl (DPPH), sodium chloride, bovine serum, hexadecyltrimethylammonium bromide (CTAB), hyaluronic acid (HA)), dissolution studies (phosphate buffer), and bioadhesive tests (mucin from porcine stomach) were obtained from Sigma-Aldrich (Poznan, Poland).

### 2.3. Extract Preparation

Ultrasonic-assisted extraction was used to prepare the orange peel extract according to the previously optimized parameters: 70% of methanol in the extraction mixture, a temperature of 70 °C, and 4 cycles per 20 min [15]. The extract was characterized in terms of the content of 11 polyphenols (catechin, caffeic acid, epicatechin, rutin, naringin, hesperidin, luteolin, quercetin, naringenin, kaempferol, hesperetin), total polyphenol content, and biological activity.

### 2.4. Extract–Chitosan Systems Preparation

The design of experiments (DoE) approach was used to prepare the extract–chitosan systems. Chitosan with different molecular weights (CS LMW, CS MMW, and CS HMW), the amount of extract in relation to CS, and the content of hypromellose (as a stabilizing substance) were used as input factors (Table 1).

The weighed ingredients were ground in an agate mortar for 60 min until a homogeneous mixture was obtained. The obtained powder mixtures were assessed in accordance with the requirements of the European Pharmacopoeia regarding bulk density, tapped density, and angle of repose.

In the DoE model, tensile strength, porosity, release, swelling index, mucoadhesive properties, as well as biological (antioxidant and anti-inflammatory) activities were used as the output parameters.

#### Identification of Obtained Systems—Infrared Spectroscopy with Attenuated Total Reflectance (IR-ATR)

Under absorbance mode, IR-ATR analysis was carried out in the 400–4000 cm^−1^ range and with a resolution of 1 cm^−1^. Lab-Solutions IR software (version 1.86 SP2) was used to run an IRTracer-100 spectrophotometer made by Shimadzu (Kyoto, Japan) to obtain the spectrogram. An attachment called QATR-10 single bounce diamond extended range was fitted to the spectrophotometer.

### 2.5. Tableting Process

Flat-faced, 8 mm in diameter tablets were compressed using a Natoli NP-RD10 (Natoli, Saint Charles, MO, USA) laboratory scale single punch tablet press. The compaction properties of tablets were assessed using several different compaction pressures in the range of 78 to 120 MPa. Pressure was released 60 s after the desired compaction pressure was achieved.

#### 2.5.1. Tablet Characterization

Tensile strength, solid fraction and porosity of tablets, weight, thickness and diameter of tablets, and hardness of tablets were measured using the method described previously [17].

#### 2.5.2. Dissolution Studies

The dissolution characteristics of tablets were examined using an Agilent 708-DS dissolving device (Agilent, Santa Clara, CA, USA). The standard basket method was used, with stirring at 37 ± 0.5 °C and 50 rpm. After adding the tablets to 200 mL of artificial saliva solution, 1 M HCl was used to bring the pH down to 6.8. Liquid samples were taken at predetermined intervals, and the same volume of temperature-adjusted medium was added. The samples were filtered using a 0.45 μm-diameter nylon membrane filter. The previously indicated HPLC method was used to determine the amounts of hesperidin in the acceptor solutions [15]. Sink conditions were maintained throughout the study. The study was repeated six times.

To investigate the release kinetics, the resulting active compound release patterns were fitted to zero-order, first-order, Higuchi, and Korsmeyer–Peppas models [18].

#### 2.5.3. Swelling Index

The water sorption aptitude of the artificial saliva solution (pH = 6.8) was used to evaluate the swellability of the tablets. After careful weighing (*W_d_*), each dry tablet was put in a beaker that was filled with solvent. At certain intervals of time (15 min, 30 min, 1 h, 2 h, 4 h, 6 h, and 24 h), the samples were placed on filter paper to remove excess surface water, and they were then weighed once more (*W_f_*). Every experiment was carried out three times. The swelling ratio was calculated according to the following equation:

$$Swelling\;index\;\left(\%\right)=\frac{\left(W_{f}-W_{d}\right)}{W_{d}}\times 100$$

#### 2.5.4. Mucoadhesive

By assessing the bonding strength between the mucin and the used polymers in the tablets, the mucoadhesive qualities of the formulations were assessed. The study used the viscosimetric technique and modified the methodology described by Hassan and Gallo [19]. The viscosity of the formulations was measured at rates of shear of 1, 5, 10, 20, 50, and 100 1/s, using the viscometer Brookfield DV2T (Brookfield, Middleborough, MA, USA).

#### 2.5.5. Biological (Antioxidant and Anti-Inflammatory) Activity of Tablets

A 2,2-Diphenyl-1-picrylhydrazyl (DPPH) assay was used to measure antioxidant activity with methods previously published [20]. A 96-well plate was filled with 25.0 μL of the tested tableting blends and 175.0 μL of DPPH solution (0.2 mM in methanol). The plates were left at room temperature for 30 min while being shaken at 300 rpm in the dark. The absorbance was measured at λ = 517 nm using a plate reader (Multiskan GO 1510, Thermo Fisher Scientific, Vantaa, Finland). IC_50_ values ± SD are used to express the results.

Anti-inflammatory activity, expressed as hyaluronidase inhibition, was determined by the turbidimetric method [20]. In short, the well was filled with 25.0 µL of incubation buffer, 25.0 µL of enzyme solution (30 U/mL), 10.0 µL of the tested tableting blends, and 15.0 µL of acetate buffer. Following a 15 min shaking incubation period at 37 °C, 25.0 µL of hyaluronic acid (HA) solution was added, and the mixture was shaken for 45 min at 37 °C. A CTAB solution in 2% sodium hydroxide (200.0 µL) was then added. A plate reader (Multiskan GO 1510, Thermo Fisher Scientific, Vantaa, Finland) was used to measure the absorbance (λ = 600 nm) following 10 min of incubation at room temperature without shaking. IC_50_ values ± SD are used to express the results.

#### 2.5.6. Microbiological Activity

Microbiological activity was assessed by determining minimal inhibitory concentration (MIC) values, as described previously [21]. To reach final concentrations of 100, 50, 25, and 12.5 mg/mL in the wells, the tableting blend of optimized formulation was serially diluted in the proper medium. A final concentration of 10^5^ CFU/mL for bacteria and 10^4^ CFU/mL for fungi was achieved by adjusting the inoculums. MIC values were then ascertained by visual analysis after the plates were incubated for 24 to 48 h at 37 °C. Color reactions were employed to enhance reading: resazurin (Merck, Warsaw, Poland) for anaerobic periopathogens and 2,3,5-triphenyltetrazolium chloride (TTC) (Sigma, Poznań, Poland) for aerobic pathogens.

### 2.6. Statistical Analysis

Statistical analysis was performed using Statistica 13.3. The Shapiro–Wilk test was used to determine whether the results were normal. Using the post hoc Tukey’s range test for multiple comparisons and the ANOVA test, the differences between the mean values were examined. At *p* < 0.05, differences across groups were deemed significant. Principal component analysis (PCA) and PQStat Software version 1.8.4.142 (2022) were used to analyze correlations.

## 3. Results and Discussion

### 3.1. Extract–Chitosan Systems Preparation

In classical drug-design research, only one element can be modified at a time and all other variables must remain constant. The statistical design of experiments (DoE) concept, a multivariate matrix-based method, measures interaction effects and covers the entire multivariate experimental area [22,23]. This statistical tool allows for the identification of factors that may be responsible for potentially the best properties of tablets rich in orange peel extract and having the highest biological activity, useful in the treatment of oral cavity infections.

For this purpose, it was decided to use orange peel extract with proven antioxidant, anti-inflammatory, and antimicrobial properties [15], directly influencing the pathogenesis of oral infections. Chitosan (CS) was added to the extract, for which it was previously shown that molecular weight (MW) has a statistically significant impact on its biological activities [10]. Therefore, in this study, the influence of the CS MW, and the ratio of extract to CS in the tableting process and the properties of the obtained tablets were assessed. Additionally, in order to enhance the mucoadhesive effect [24], the impact of HPMC on the process was assessed. Therefore, according to the experiment matrix (Table 1), nine tableting blends were prepared and further evaluated.

**Figure 1:**
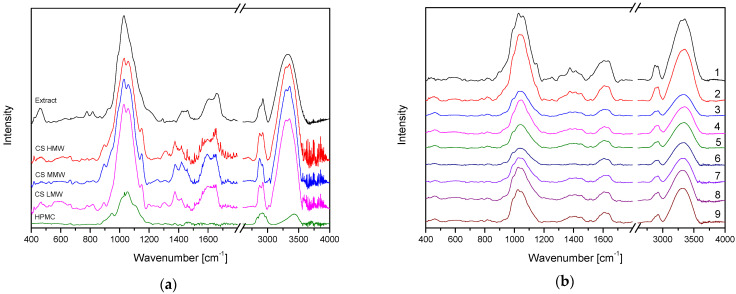
ATR-IR spectra for orange peel extract [15], CS [26], and HPMC [8] (**a**), and formulation F1–F9 (**b**).

Before examining the physicochemical properties of all formulations, the mixtures were assessed for intermolecular interactions (Figure 1). A detailed analysis of the extract spectrum was performed in an earlier work, where the occurrence of hesperidin bands in the extract was assessed [15]. In brief, the spectrum peak of carbonyl C = O stretch appeared at 1645 cm^−1^. The bands at 1518–1356 cm^−1^ are attributed to the aromatic C = C stretch and the aromatic C–O stretch at 1273, 1204, 1150, 1126, and 1067 cm^−1^. It is easy to identify the characteristic absorption bands in the CS spectrum: strong absorption bands at 1150 cm^−1^ and 1059 cm^−1^ due to the asymmetric stretching of the C-O–C bond and the C–O stretching vibration of secondary alcohols, respectively; a broad band around 3300 cm^−1^ attributed to the overlapped peaks of the O–H and N–H stretching vibrations, the amide I (C = O stretching) and amide II (N-H bending) modes at 1638 and 1578 cm^−1^, respectively [25,26]. A broad band at 3300 cm^−1^ was seen in the HPMC spectrum, which is linked to the presence of –OH groups. Several C–O vibrations, such as glycosidic C–O–C, C–OH, C–OCH_3_, and C–OCH_2_CH_2_OH, are associated with the complex band between 1200 and 950 cm^−1^ [8,27]. In the case of tableting blends, the spectrum is the sum of the spectra of the suspension components.

Then, the properties of the powders for tableting were evaluated, where first, flow properties were checked (Table 2).

The significance of the input parameters on true density is shown in Figure S1 (Supplementary Material). It was noticed that there was a statistically significant effect of extract and HPMC content on true density; as the extract content increases and HPMC decreases, the true density increases.

### 3.2. Tableting Process

**Figure 2:**
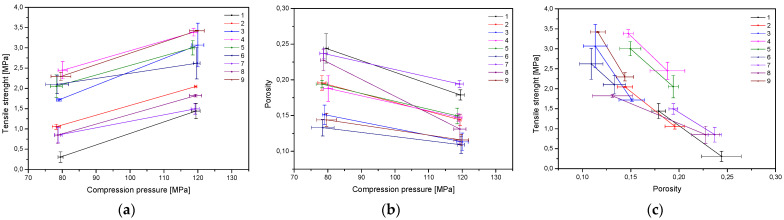
Tabletability (**a**), compressibility (**b**), and compactibility (**c**) profiles of tablets produced from systems 1–9.

All formulations were tableted correctly, and the process was reproducible. Tablet characterization consisted of tabletability, compressibility, and compactibility (Figure 2) [17]. At low compaction pressures, the formulation F4 proved its capacity to create the toughest tablets (Figure 1a). Compressibility was the highest for formulation F1, because a relatively high porosity was retained at higher compression pressure values (Figure 1b). Based on the above-mentioned parameters, the tablets produced from formulation F4 have better properties, since they demonstrated good tabletability (the strongest tablets at the lowest compaction pressures), good compressibility, as well as higher compactibility, compared to the other tablets (the strongest tablet with the highest solid fraction).

The significance of the input parameters on tablets’ porosity is shown in Figure S2 (Supplementary Material). It was noticed that there was a statistically significant effect of extract content on tablets’ porosity; as the extract content increases, the porosity decreases.

**Figure 3:**
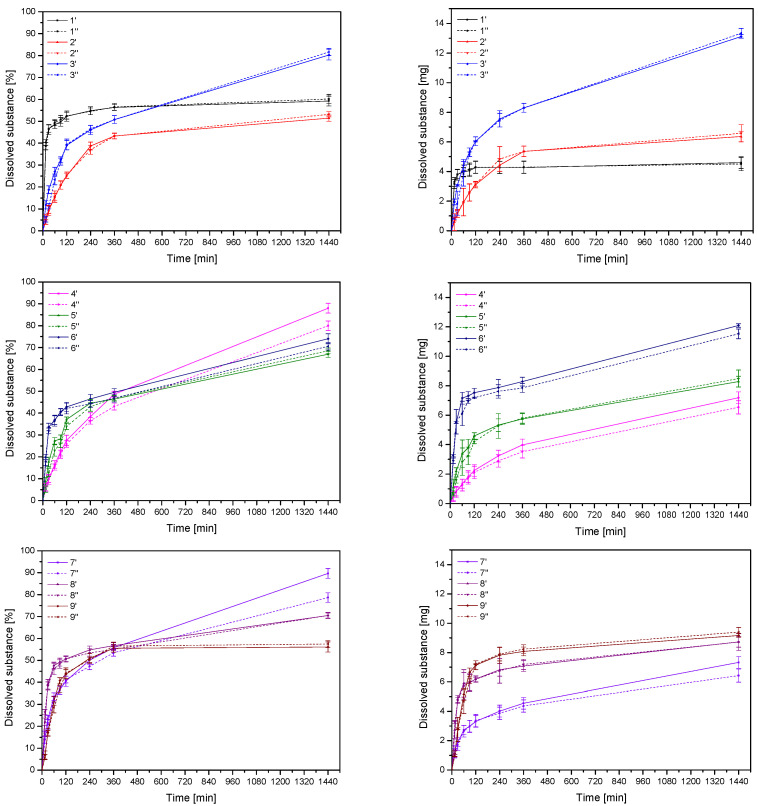
Hesperidin dissolution profiles for tablets F1–F9 for both compression pressures (′ for lower compression pressure and ″ for higher compression pressure).

Dissolution is a crucial pharmaceutical property, as it directly impacts bioavailability and the provision of the desired therapeutic effects to patients. So, the release kinetics of hesperidin from all formulations, expressed in % and mg, were determined (Figure 3). The dissolution profiles for the same formulation but prepared by different compression pressure did not differ statistically (*f*_1_ below 15 and *f*_2_ above 50, close to 100) (Table S1, Supplementary Materials). The comparison of the release profiles of individual formulations is summarized in Tables S2 and S3 (Supplementary Materials). Analyzing the Pareto chart regarding the amount of released hesperidin, the amount of extract within formulations is a factor that statistically significantly determines the release (Figure S3, Supplementary Materials). On the other hand, increased amounts of HPMC in formulations delay the release of hesperidin (Figure S3, Supplementary Materials). This phenomenon can be explained by the fact that HPMC swells, creating a gel matrix that controls the release of the drug [28].

To identify the mechanism causing the release of the hesperidin, the dissolution data obtained for all formulations were fitted to the release models of zero-order and first-order equations, the Higuchi model (used for the matrix systems), and the Korsmeyer–Peppas model (used for the swellable matrices) (Table S4, Supplementary Materials). Kinetic simulations that examined all formulations indicate that the tablets mostly adhere to the Higuchi model. According to this hypothesis, the drug was mainly released by a diffusion mechanism, whereby it was released from a homogenous planar matrix [29].

**Figure 4:**
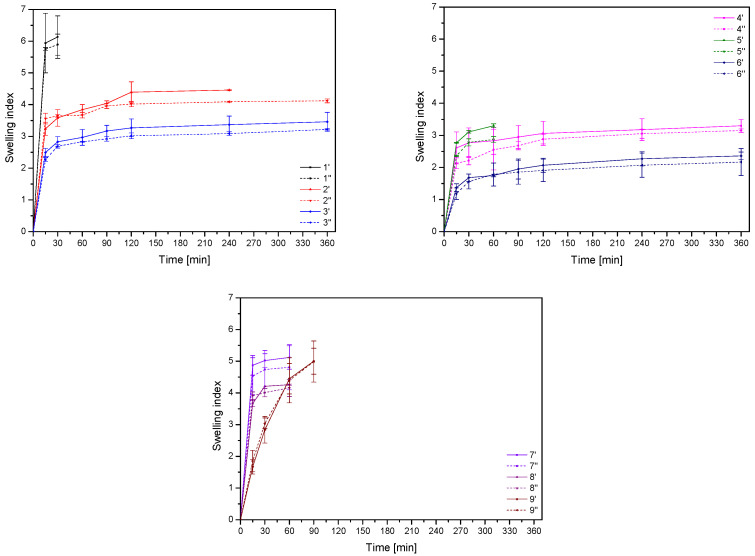
Swelling index for tablets F1–F9 for both compression pressures (′ for lower compression pressure and ″ for higher compression pressure).

The above phenomenon can also be observed by analyzing the swelling index (Figure 4). However, in this case the relationships are not statistically significant (Figure S4, Supplementary Materials). So, swelling studies were carried out to confirm the drug delivery mechanism. In the case of formulations 1, 5, 7, 8 and 9, higher release can be attributed to the higher level of water uptake, which leads to the enhanced wetting and penetration of water into the matrix, consequently increasing diffusion of the active substance, while the tablets disintegrated within 90 min. The rapid disintegration of the tablets disqualifies them from the prolonged stay at the site of application necessary to produce a prolonged therapeutic effect. 

In the release studies, hesperitin, the aglycone of hesperidin, was additionally monitored, the concentration of which was proportional to the main analyte. No additional peaks were observed in the chromatogram indicating hesperidin degradation products in the tested medium, which allows us to draw conclusions on the stability of hesperidin.

**Figure 5:**
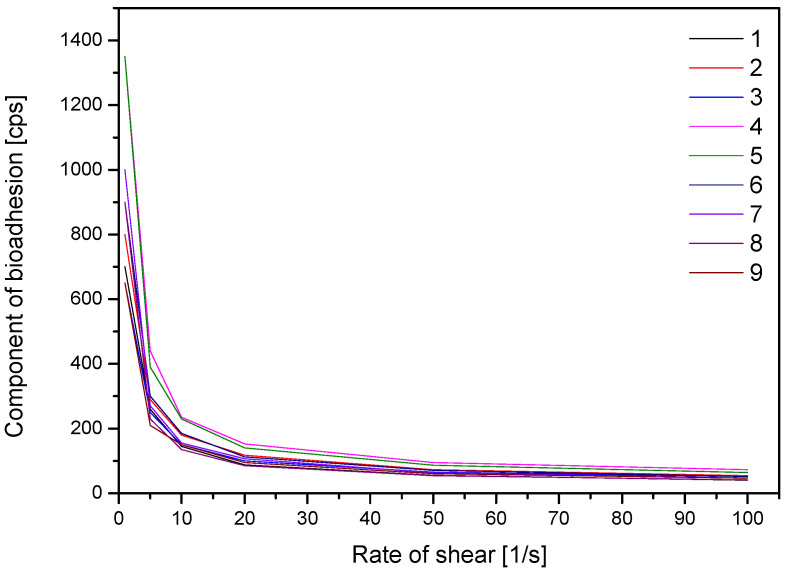
Component of bioadhesion of formulations F1–F9.

A crucial element influencing the buccal preparation’s application characteristics is the polymer’s capacity to attach to mucin in the mucosa. This guarantees that the product stays on the oral mucosa and prevents it from being swallowed into the digestive system. Rheological testing of formulation blends was performed for this purpose; Figure 5 shows the findings. Based on the Pareto chart, it was noticed that an increase in extract concentration results in a decrease in mucoadhesive properties (Figure S5, Supplementary Materials). On the other hand, the increase in the concentration of HPMC and CS caused an increase in the formulations’ mucoadhesive properties; however, these relationships are not statistically significant. Both biopolymers are known for their widely described bioadhesive properties and therefore were included in this study [30,31].

Finally, biological activity studies were also performed for tableting blends (antioxidant and anti-inflammatory activity tests). Orange peel, as well as hesperidin as a main active component, has been recently extensively evaluated for its health-promoting effects to reveal its e.g., antioxidant and anti-inflammatory activities [15]. This activity was also proven in the formulation blends (Table 3). As expected, as the extract content in the mixtures increases, their antioxidant activity increases (Figure S6, Supplementary Materials). CS is mainly responsible for the anti-inflammatory activity, expressed as the ability to inhibit the activity of the hyaluronidase enzyme [10] (Figure S7, Supplementary Materials).

It is worth correlating the obtained values for the formulation with the level of hesperidin release in the availability studies. The content of hesperidin in the lyophilized extract was 23 μg/1 mg of extract. Antioxidant activity (Table 3) IC_50_ = 2 mg of extract/mL, which corresponds to 46 μg of hesperidin/mL. Analyzing for example the concentration of the substance released from formulation 3 within 6 h, it is 8 mg, which gives a concentration of 40 μg/mL, which is in accordance with the IC_50_, so it is still a guarantee of the activity of the formulation.

**Figure 6:**
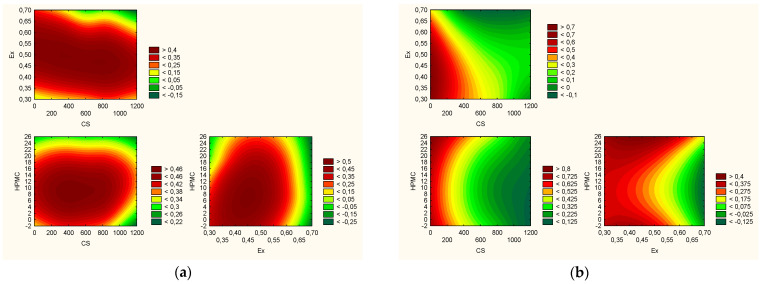
Prediction based on effects with positive signs (true density, porosity, dissolution after 6 h expressed in mg, swelling index, component of mucoadhesion) (**a**) and with negative signs (antioxidant and anti-inflammatory activities) (**b**).

For every effect that was investigated, a utility contour model of the profiles was created (Figure 6). The finest characteristics were found in the formulation with the composition of CS HMW, extract content of 60%, and HPMC content of 8%, based on the examination of the accompanying charts. They fall halfway between the greatest mechanical qualities brought about by the high biopolymers’ concentration and the best chemical and biological features brought about by the high extract content.

**Figure 7:**
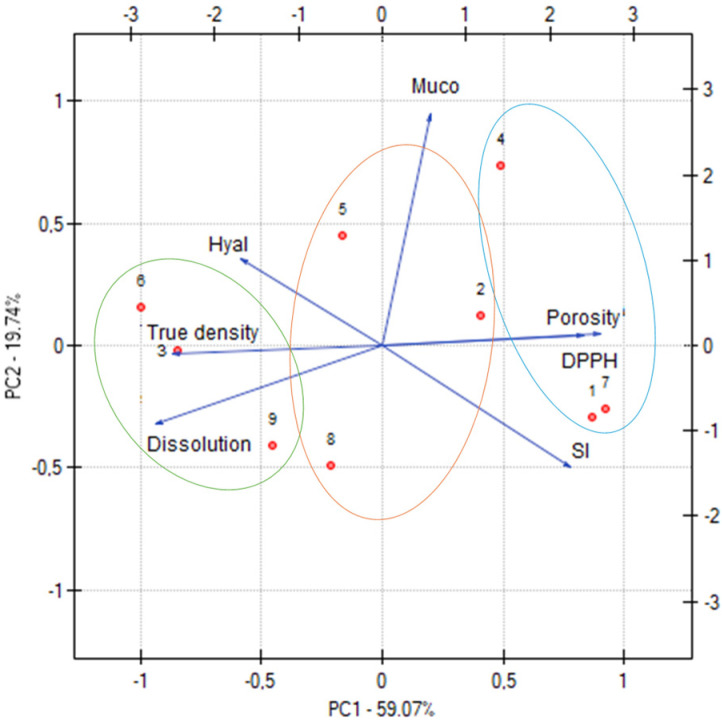
Principal component analysis (PCA) showing the factor loading plot considering true density, porosity, dissolution after 6 h expressed in mg, swelling index, component of mucoadhesion (=Muco), and antioxidant (=DPPH) and anti-inflammatory activities (=Hyal).

To analyze the relationship between the effects, principal component analysis (PCA) was performed, which is a popular data analysis method that allows for the observation of regularities between the studied variables. Figure 7 as well as Table S5 (Supplementary Materials) show the results of PCA analysis of the extracts, considering all formulations. A negative correlation was observed between true density and porosity. A strong negative correlation was noticed between porosity and dissolution, which can be explained by the fact that the release mechanism is such that the substance is released from the matrix without any structural changes that could occur if porosity was involved in the release process; this also explains the negative correlation between dissolution and swelling index. A negative correlation was also noted between antioxidant activity, mainly mediated by the extract content, and anti-inflammatory activity associated with CS, as described above.

Based on the PCA plot (Figure 7), it was possible to divide all formulations into three groups. This division corresponds to the extract content in the formulation blends. The blue line surrounds the formulations with the lowest extract content, the orange line surrounds the formulations with intermediate content, and the green line surrounds the formulations with the highest extract content. The graph confirms previous findings that hesperidin release was closely related to extract content, as described above.

For the optimized formulation, additional tests were performed on microbiological activity against bacteria responsible for oral infections. According to numerous studies, CS by itself possesses strong antibacterial qualities against a variety of microorganisms, including *S. aureus*, *E. coli*, etc. [32]. Its antimicrobial activity is still unclear due to the complexity of its action on microorganisms, so there are several hypotheses regarding this aspect. One of the most widely accepted theories explains how the negatively charged microbial cell membrane and the positively charged amino groups of chitosan interact electrostatically to alter their permeability and impede microbial development. The chelation of chitosan molecules with certain metal ions, which harms the cell walls of microorganisms, is another theory [33]. The optimized formulation showed broad microbiological activity against the yeast *Candida albicans*, the cariogenic bacterium *Streptococcus mutans*, and the periopathogens *Porphyromonas gingivalis*, *Fusobacterium nucleatum*, and *Actinomyces odontolyticus* (Table 4), higher than lyophilized extract and CS alone. The antimicrobial activity of flavonoids is due to their ability to complex with extracellular and soluble protein and to complex with bacterial cells [34]. The differences in the fluctuations of antimicrobial activity could be due to the synergistic mechanism of action of CS and polyphenols contained in the extract. Activity against the bacteria responsible for wound infections *Staphylococcus aureus* and *Pseudomonas aeruginosa*, was comparable. Additionally, the results obtained for formulations were compared with an oral antifungal nystatin and an amoxicillin antibiotic as a positive control (Table 4). The activity of these compounds was greater than that of the extract. However, the proposal to use the extract and optimized formulation in the treatment of oral infections is not based on isolated antimicrobial activity, but on the whole spectrum of activity, from antioxidant and anti-inflammatory to antimicrobial.

## 4. Conclusions

The use of a statistical design of experiments (DoE) enabled the identification of key factors influencing the tablet’s mechanical and biological properties and is a useful approach in modern pharmaceutical development. In the presented case study, the finest characteristics were found in the formulation with the composition of CS HMW, extract content of 60%, and HPMC content of 8%. Overall, the optimized formulation combining high CS molecular weight, substantial orange peel extract, and adequate HPMC content offers a promising approach for developing effective buccal tablets with enhanced mechanical properties, controlled release, and significant antimicrobial and anti-inflammatory activities. This formulation has potential clinical applications in the treatment of oral cavity infections, providing a foundation for further development and in vivo studies. Future trends in buccal tablet development are likely to focus on improving patient compliance, enhancing drug efficacy through novel materials and delivery systems, and leveraging digital technologies to optimize formulation processes and therapeutic outcomes.

## Figures and Tables

**Table 1:** Compositions of extract–chitosan systems.

	CS [MW Expressed as Average cps]	Extract [Amount in Relation to CS]	Hypromellose [%]
F1	100	0.33	0
F2	100	0.5	25
F3	100	0.66	12.5
F4	600	0.33	25
F5	600	0.5	12.5
F6	600	0.66	0
F7	1100	0.33	12.5
F8	1100	0.5	0
F9	1100	0.66	25

CS—chitosan, MW—molecular weight.

**Table 2:** Comparison of Systems’ Flowability Properties.

	True Density [g/cm^3^]	Bulk Density [g/cm^3^]	Hausner Ratio	Compactability Index [%]	Angle of Repose [°]
F1	1.47± 0.01	0.53 ± 0.01	1.46 ± 0.01	31.58 ± 0.01	39.10 ± 1.42
F2	1.46 ± 0.01	0.67 ± 0.01	1.15 ± 0.01	13.33 ± 0.01	38.04 ± 0.81
F3	1.48 ± 0.01	0.67 ± 0.01	1.36 ± 0.01	26.67 ± 0.01	31.26 ± 1.26
F4	1.46 ± 0.01	0.34 ± 0.01	1.42 ± 0.05	29.31 ± 2.43	54.89 ± 1.56
F5	1.48 ± 0.01	0.42 ± 0.01	1.30 ± 0.05	22.92 ± 2.94	46.33 ± 2.12
F6	1.49 ± 0.01	0.50 ± 0.01	1.21 ± 0.05	17.50 ± 3.54	46.01 ± 2.16
F7	1.46 ± 0.01	0.50 ± 0.01	1.25 ± 0.01	20.00 ± 0.01	46.00 ± 1.20
F8	1.48 ± 0.01	0.53 ± 0.01	1.31 ± 0.06	23.68 ± 3.72	38.62 ± 0.32
F9	1.47 ± 0.01	0.67 ± 0.01	1.25 ± 0.01	20.00 ± 0.01	38.69 ± 1.95

**Table 3:** Antioxidant and anti-inflammatory activities for formulations F1–F9.

	Antioxidant Activity IC_50_ [mg/mL]	Anti-Inflammatory Activity IC_50_ [mg/mL]
F1	30.69 ± 5.32	8.60 ± 0.35
F2	33.97 ± 11.51	13.67 ± 0.72
F3	17.16 ± 7.76	21.85 ± 0.87
F4	12.28 ± 1.39	8.07 ± 0.75
F5	8.97 ± 1.26	11.71 ± 1.00
F6	7.32 ± 0.21	14.77 ± 0.48
F7	21.08 ± 1.58	1.92 ± 0.02
F8	12.34 ± 0.64	3.72 ± 0.37
F9	9.47 ± 0.29	6.79 ± 0.23
Lyophilized extract	2.03 ± 0.18	4.43 ± 0.21

**Table 4:** Microbiological activity of CS, lyophilized extract, and optimized formulation.

	CS	Lyophilized Extract	Optimized Formulation	Positive Control	
				Nystatin	Amoxicillin
	MIC [mg/mL]			MIC [μg/mL]	
*Staphylococcus aureus*	>100	>100	>100		0.06 [35]
*Pseudomonas aeruginosa*	>100	>100	>100		na [36]
*Candida albicans*	>100	>100	100	0.5 [37]	
*Streptococcus mutans* ATCC 25175	>100	>100	100		<0.10 [38]
*Porphyromonas gingivalis* ATCC 33277	100	>100	12.5		<0.02 [39]
*Prevotella intermedia* ATCC 25611	100	>100	25		
*Fusobacterium nucleatum* ATCC 25586	100	>100	25		0.02 [40]
*Schaalia odontolytica* (*Actinomyces odontolyticus*) ATCC 17929	100	>100	12.5		0.19 [41]

CS—chitosan, MIC—minimum inhibitory concentration, na—non active.

## Data Availability

The original contributions presented in the study are included in the article/Supplementary Material, further inquiries can be directed to the corresponding authors.

## Supplementary Materials

The following supporting information can be downloaded at: https://www.mdpi.com/article/10.3390/ma17215234/s1. Figure S1. Statistical analysis of true density of systems 1–9: (a) Pareto plot of standardized effects on true density of systems 1–9; (b) Response surface plots presenting the dependence of extract content and HPMC content on the true density for constant CS MW of 600 cps. Figure S2. Statistical analysis of tablets’ porosity: (a) Pareto plot of standardized effects on tablets’ porosity; (b) Response surface plots presenting the dependence of extract content and CS MW on the tablets’ density for constant HPMC content of 12.5%. Table S1. Comparison of profiles within the same formulation between different compression forces (a) expressed in% and (b) in mg. Table S2. Comparison of profiles of different formulations for (a) lower and (b) higher compression forces, expressed in%. Table S3. Comparison of profiles of different formulations for (a) lower and (b) higher compression forces, expressed in mg. Figure S3. Statistical analysis of tablets’ dissolution after 6h expressed in mg: (a) Pareto plot of standardized effects on tablets’ dissolution; (b) Response surface plots presenting the dependence of extract content and HPMC content on the tablets’ dissolution for constant CS MW of 600 cps. Table S4. Parameters of mathematical models fitted to the chlorogenic acid release profiles of nanofibers N1–N4. Figure S4. Statistical analysis of tablets’ swelling index: (a) Pareto plot of standardized effects on swelling index; (b) Response surface plots presenting the dependence of extract content and HPMC content on swelling index for constant CS MW of 600 cps. Figure S5. Statistical analysis of component of bioadhesion: (a) Pareto plot of standardized effects on component of bioadhesion; (b) Response surface plots presenting the dependence of extract and HPMC content on the formulations’ component of bioadhesion for constant CS MW of 600 cps. Figure S6. Statistical analysis of antioxidant activity: (a) Pareto plot of standardized effects on antioxidant activity; (b) Response surface plots presenting the dependence of extract content and CS MW on the formulations’ antioxidant activity for constant HPMC content of 12.5%. Figure S7. Statistical analysis of anti-inflammatory activity: (a) Pareto plot of standardized effects on anti-inflammatory activity; (b) Response surface plots presenting the dependence of CS MW and extract content on the formulations’ anti-inflammatory activity for constant HPMC content of 12.5%. Table S5. Correlation matrix.
