# Supplementary material for: Implementing the Design of Experiments (DoE) Concept into the Development of Mucoadhesive Tablets Containing Orange Peel Extract as a Potential Concept for the Treatment of Oral Infections
Source: Materials (Basel). 2024 Oct 28;17(21):5234. doi: 10.3390/ma17215234 (PMC11547214; doi:10.3390/ma17215234)
Supplement: Supplementary file 1 [file materials-17-05234-s001.zip › materials-3240994-supplementary.pdf]

Implementing the Design of Experiments (DoE) Concept into the Development of Mucoadhesive Tablets Containing Orange Peel Extract as a Potential Concept for the Treatment of Oral Infections

Supplementary Material

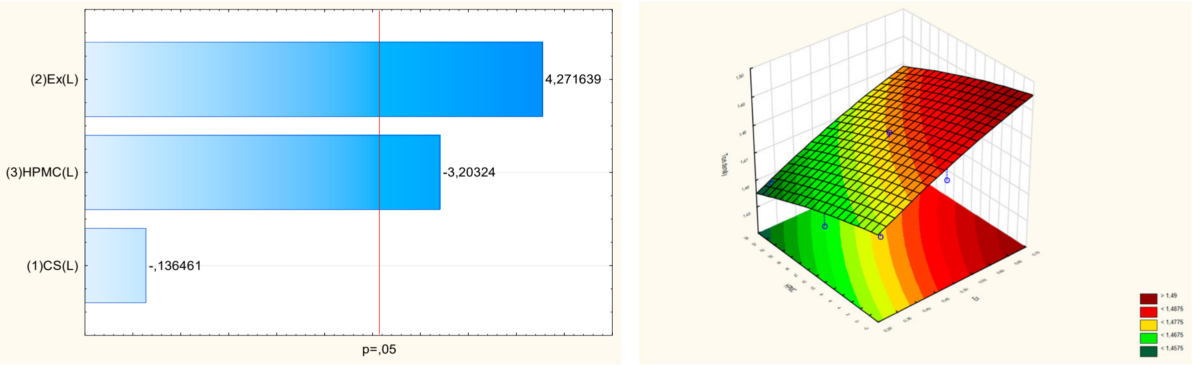

(a) (b)

Figure S1. Statistical analysis of true density of systems 1–9: (a) Pareto plot of standardized effects on true density of systems 1-9; (b) Response surface plots presenting the dependence of extract content and HPMC content on the true density for constant CS MW of 600 cps.

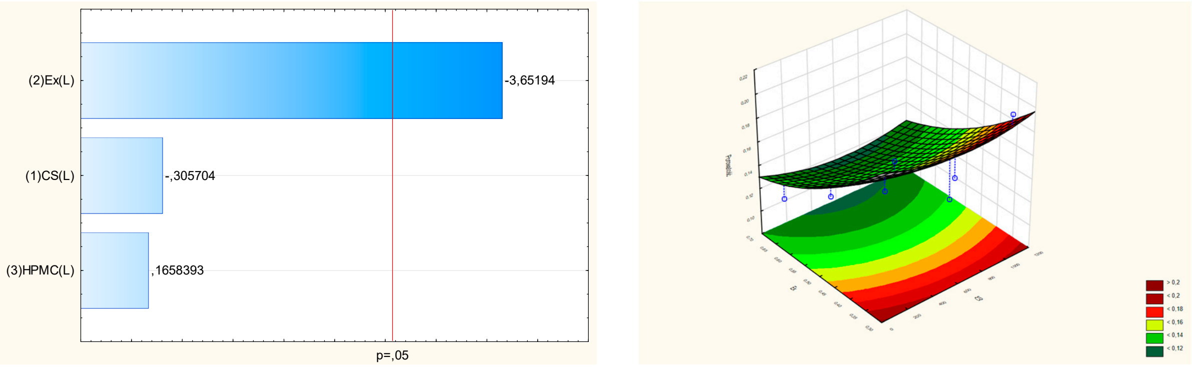

(a) (b)

Figure S2. Statistical analysis of tablets' porosity: (a) Pareto plot of standardized effects on tablets' porosity; (b) Response surface plots presenting the dependence of extract content and CS MW on the tablets' density for constant HPMC content of 12.5 %.

Table S1. Comparison of profiles within the same formulation between different compression forces (a) expressed in % and (b) in mg.

(a)

	1′	2′	3′	4′	5′	6′	7′	8′	9′
1″	f_1 = 1.46<br>f_2 = 98.77								
2″		f_1 = 3.92<br>f_2 = 78.34							
3″			f_1 = 6.76<br>f_2 = 62.74						
4″				f_1 = 7.98<br>f_2 = 59.72					
5″					f_1 = 7.82<br>f_2 = 61.39				
6″						f_1 = 3.82<br>f_2 = 75.53			
7″							f_1 = 6.66<br>f_2 = 56.91		
8″								f_1 = 2.72<br>f_2 = 96.69	
9″									f_1 = 3.80<br>f_2 = 82.82

(b)

	1′	2′	3′	4′	5′	6′	7′	8′	9′
1″	f_1 = 1.40<br>f_2 = 99.90								
2″		f_1 = 3.77<br>f_2 = 98.99							
3″			f_1 = 6.71<br>f_2 = 92.86						
4″				f_1 = 8.80<br>f_2 = 96.89					
5″					f_1 = 8.24<br>f_2 = 94.91				
6″						f_1 = 5.74<br>f_2 = 94.10			
7″							f_1 = 6.39<br>f_2 = 97.02		
8″								f_1 = 2.23<br>f_2 = 99.85	
9″									f_1 = 3.82<br>f_2 = 99.30

Table S2. Comparison of profiles of different formulations for (a) lower and (b) higher compression forces. expressed in %.

(a)

	1′	2′	3′	4′	5′	6′	7′	8′	9′
1′									
2′	f_1 = 48.79<br>f_2 = 8.91								
3′	f_1 = 35.23<br>f_2 = 23.50	f_1 = 46.63<br>f_2 = 24.41							
4′	f_1 = 51.12<br>f_2 = 14.90	f_1 = 23.31<br>f_2 = 39.76	f_1 = 21.16<br>f_2 = 39.11						
5′	f_1 = 35.87<br>f_2 = 17.99	f_1 = 32.58<br>f_2 = 32.19	f_1 = 9.83<br>f_2 = 50.36	f_1 = 26.00<br>f_2 = 58.48					
6′	f_1 = 23.47<br>f_2 = 32.78	f_1 = 63.56<br>f_2 = 17.69	f_1 = 16.52<br>f_2 = 46.35	f_1 = 43.91<br>f_2 = 27.42	f_1 = 23.37<br>f_2 = 33.28				
7′	f_1 = 30.14<br>f_2 =34.17	f_1 = 65.54<br>f_2 = 17.03	f_1 = 12.89<br>f_2 = 42.97	f_1 = 34.62<br>f_2 = 26.39	f_1 = 24.86<br>f_2 = 31.94	f_1 = 13.69<br>f_2 = 88.44			
8′	f_1 = 9.94<br>f_2 = 61.49	f_1 = 86.93<br>f_2 = 10.90	f_1 = 33.90<br>f_2 = 27.58	f_1 = 65.66<br>f_2 = 17.56	f_1 = 41.00<br>f_2 = 21.09	f_1 = 16.35<br>f_2 = 39.38	f_1 = 24.02<br>f_2 = 41.29		
9′	f_1 = 25.82<br>f_2 = 22.72	f_1 = 44.85<br>f_2 = 25.26	f_1 = 18.87<br>f_2 = 89.92	f_1 = 42.65<br>f_2 = 40.80	f_1 = 18.81<br>f_2 = 53.26	f_1 = 18.13<br>f_2 = 44.20	f_1 = 17.23<br>f_2 = 42.03	f_1 = 22.51<br>f_2 = 26.64	

(b)

	1′′	2′′	3′′	4′′	5′′	6′′	7′′	8′′	9′′
1′′									
2′′	f_1 = 48.28<br>f_2 = 9.08								
3′′	f_1 = 39.59<br>f_2 = 20.08	f_1 = 38.05<br>f_2 = 29.11							
4′′	f_1 = 51.51<br>f_2 = 12.21	f_1 = 13.53<br>f_2 = 52.57	f_1 = 18.61<br>f_2 = 38.03						
5′′	f_1 = 40.64<br>f_2 = 15.13	f_1 = 23.78<br>f_2 = 39.75	f_1 = 11.64<br>f_2 = 49.58	f_1 = 19.61<br>f_2 = 57.00					
6′′	f_1 = 23.66<br>f_2 = 29.81	f_1 = 57.43<br>f_2 = 19.63	f_1 = 26.76<br>f_2 = 42.12	f_1 = 47.85<br>f_2 = 24.96	f_1 = 28.34<br>f_2 = 30.54				
7′′	f_1 = 29.75<br>f_2 = 27.45	f_1 = 53.31<br>f_2 = 21.25	f_1 = 13.88<br>f_2 = 47.02	f_1 = 37.38<br>f_2 = 27.05	f_1 = 24.95<br>f_2 = 33.28	f_1 = 13.24<br>f_2 = 75.60			
8′′	f_1 = 9.95<br>f_2 = 58.39	f_1 = 83.82<br>f_2 = 11.42	f_1 = 41.81<br>f_2 = 24.11	f_1 = 71.41<br>f_2 = 14.93	f_1 = 49.81<br>f_2 = 18.28	f_1 = 16.84<br>f_2 = 36.49	f_1 = 24.97<br>f_2 = 33.35		
9′′	f_1 = 27.43<br>f_2 = 21.35	f_1 = 40.57<br>f_2 = 27.31	f_1 = 19.03<br>f_2 = 80.30	f_1 = 43.87<br>f_2 = 35.37	f_1 = 22.91<br>f_2 = 45.23	f_1 = 21.45<br>f_2 = 45.84	f_1 = 14.35<br>f_2 = 51.77	f_1 = 24.43<br>f_2 = 25.65	

Table S3. Comparison of profiles of different formulations for (a) lower and (b) higher compression forces. expressed in mg.

(a)

	1′	2′	3′	4′	5′	6′	7′	8′	9′
1′									
2′	f_1 = 40.21<br>f_2 = 79.63								
3′	f_1 = 65.64<br>f_2 = 61.79	f_1 = 94.99<br>f_2 = 54.48							
4′	f_1 = 51.18<br>f_2 = 70.15	f_1 = 23.81<br>f_2 = 87.47	f_1 = 57.60<br>f_2 = 50.88						
5′	f_1 = 34.41<br>f_2 = 96.52	f_1 = 34.89<br>f_2 = 75.22	f_1 = 30.82<br>f_2 = 63.17	f_1 = 63.17<br>f_2 = 67.10					
6′	f_1 = 82.52<br>f_2 = 52.88	f_1 = 99.00<br>f_2 = 47.74	f_1 = 22.17<br>f_2 = 75.15	f_1 = 78.23<br>f_2 =45.04	f_1 = 70.51<br>f_2 = 54.45				
7′	f_1 = 31.89<br>f_2 = 87.64	f_1 = 20.15<br>f_2 = 93.62	f_1 = 43.32<br>f_2 = 56.98	f_1 = 33.69<br>f_2 = 79.50	f_1 = 19.58<br>f_2 = 81.91	f_1 = 51.95<br>f_2 = 49.55			
8′	f_1 = 51.49<br>f_2 = 63.73	f_1 = 89.05<br>f_2 = 55.86	f_1 = 22.50<br>f_2 = 97.53	f_1 = 98.68<br>f_2 = 52.05	f_1 = 40.15<br>f_2 = 66.34	f_1 = 17.81<br>f_2 = 72.05	f_1 = 71.05<br>f_2 = 58.53		
9′	f_1 = 66.70<br>f_2 = 64.10	f_1 = 87.97<br>f_2 = 56.12	f_1 = 17.53<br>f_2 = 96.69	f_1 = 97.38<br>f_2 = 52.27	f_1 = 39.35<br>f_2 = 66.76	f_1 = 18.27<br>f_2 = 71.53	f_1 = 71.69<br>f_2 = 58.83	f_1 = 17.57<br>f_2 = 99.91	

(b)

	1″	2″	3″	4″	5″	6″	7″	8″	9″
1″									
2″	f_1 = 41.45<br>f_2 = 82.37								
3″	f_1 = 71.52<br>f_2 = 65.56	f_1 = 77.14<br>f_2 = 58.15							
4″	f_1 = 52.93<br>f_2 = 67.37	f_1 = 26.80<br>f_2 = 79.55	f_1 = 58.68<br>f_2 = 51.77						
5″	f_1 = 41.22<br>f_2 = 99.80	f_1 = 22.41<br>f_2 = 83.58	f_1 = 31.50<br>f_2 = 64.97	f_1 = 65.77<br>f_2 = 68.03					
6″	f_1 = 72.68<br>f_2 = 55.02	f_1 = 92.22<br>f_2 = 50.13	f_1 = 29.42<br>f_2 = 74.41	f_1 = 89.92<br>f_2 = 45.52	f_1 = 74.89<br>f_2 = 54.65				
7″	f_1 = 30.70<br>f_2 = 82.55	f_1 = 16.23<br>f_2 = 99.98	f_1 = 44.63<br>f_2 = 58.21	f_1 = 38.07<br>f_2 = 79.38	f_1 = 21.26<br>f_2 = 83.76	f_1 = 52.77<br>f_2 = 50.17			
8″	f_1 = 49.45<br>f_2 = 63.18	f_1 = 83.67<br>f_2 = 56.42	f_1 = 31.21<br>f_2 = 96.89	f_1 = 95.92<br>f_2 = 50.47	f_1 = 51.37<br>f_2 = 62.65	f_1 = 13.52<br>f_2 = 78.37	f_1 = 83.24<br>f_2 = 56.48		
9″	f_1 = 64.86<br>f_2 = 65.08	f_1 = 78.39<br>f_2 =57.80	f_1 = 17.87<br>f_2 = 99.87	f_1 = 93.70<br>f_2 = 51.52	f_1 = 47.01<br>f_2 = 64.51	f_1 = 18.32<br>f_2 = 75.12	f_1 = 79.21<br>f_2 = 57.87	f_1 = 19.06<br>f_2 = 97.87	

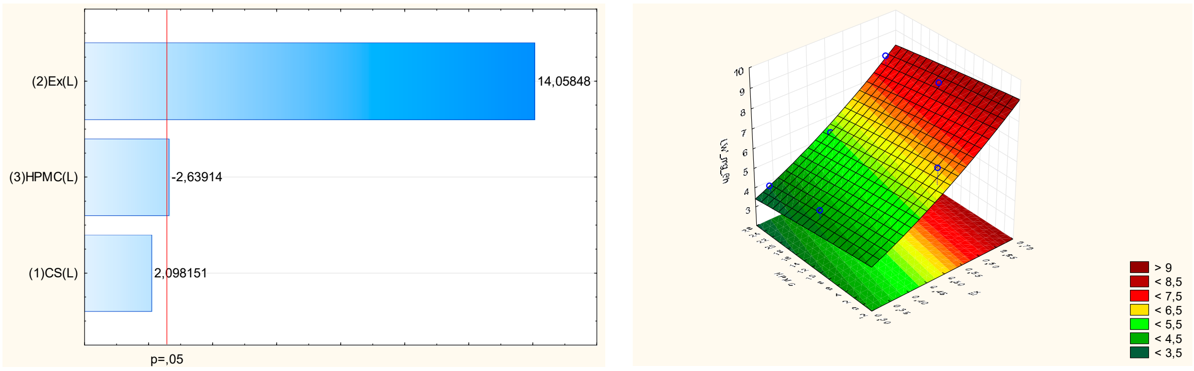

(a) (b)

Figure S3. Statistical analysis of tablets' dissolution after 6h expressed in mg: (a) Pareto plot of standardized effects on tablets' dissolution; (b) Response surface plots presenting the dependence of extract content and HPMC content on the tablets' dissolution for constant CS MW of 600 cps.

Table S4. Parameters of mathematical models fitted to the chlorogenic acid release profiles of nanofibers N1–N4.

Formulation	**Mathematical model**							
No.	**Zero-order kinetic**		**First-order kinetic**		**Higuchi kinetic**		**Korsmeyer-Peppas kinetic**	
	K	R^2	K	R^2	K	R^2	R^2	n
F1	0..02	0.18	0.01	0.07	**6.17**	**0.71**	0.55	0.41
F2	0.03	0.59	0.01	0.29	**6.00**	**0.97**	0.90	0.46
F3	0.04	0.74	0.01	0.26	**7.98**	**0.94**	0.79	0.46
F4	0.06	0.87	0.01	0.39	8.22	0.84	**0.92**	**0.49**
F5	0.04	0.65	0.01	0.24	**7.06**	**0.97**	0.80	0.45
F6	0.03	0.60	0.01	0.17	**7.20**	**0.91**	0.68	0.43
F7	0.05	0.76	0.01	0.24	**8.60**	**0.92**	0.75	0.46
F8	0.03	0.39	0.01	0.13	**7.46**	**0.88**	0.64	0.44
F9	0.03	0.34	0.01	0.18	**7.29**	**0.96**	0.79	0.47

The most fitting mathematical model is shown in bold.

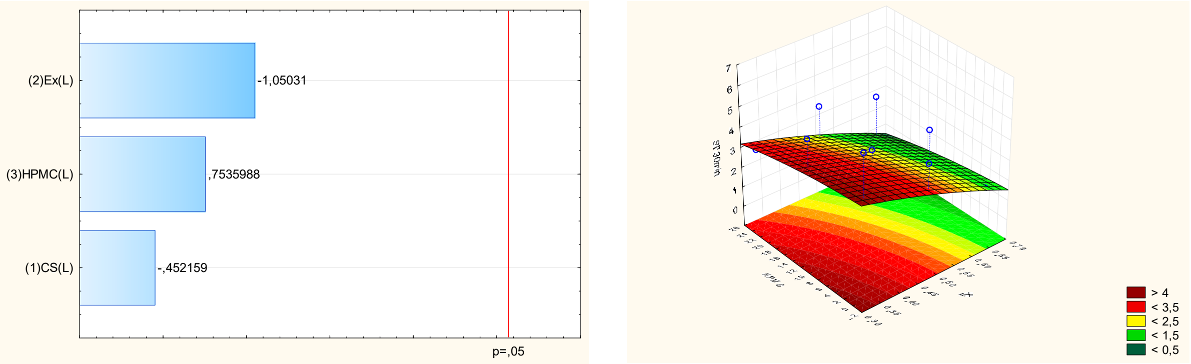

(a) (b)

Figure S4. Statistical analysis of tablets' swelling index: (a) Pareto plot of standardized effects on swelling index; (b) Response surface plots presenting the dependence of extract content and HPMC content on swelling index for constant CS MW of 600 cps.

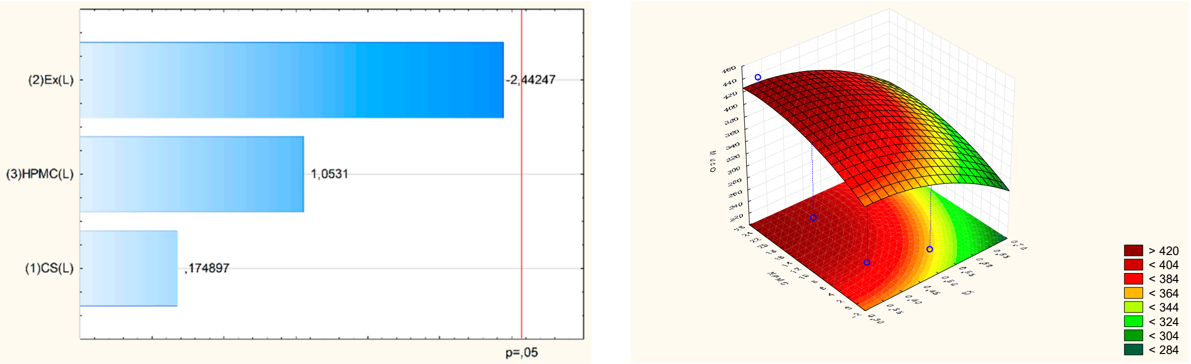

(a) (b)

Figure S5. Statistical analysis of component of bioadhesion: (a) Pareto plot of standardized effects on component of bioadhesion; (b) Response surface plots presenting the dependence of extract and HPMC content on the formulations' component of bioadhesion for constant CS MW of 600 cps.

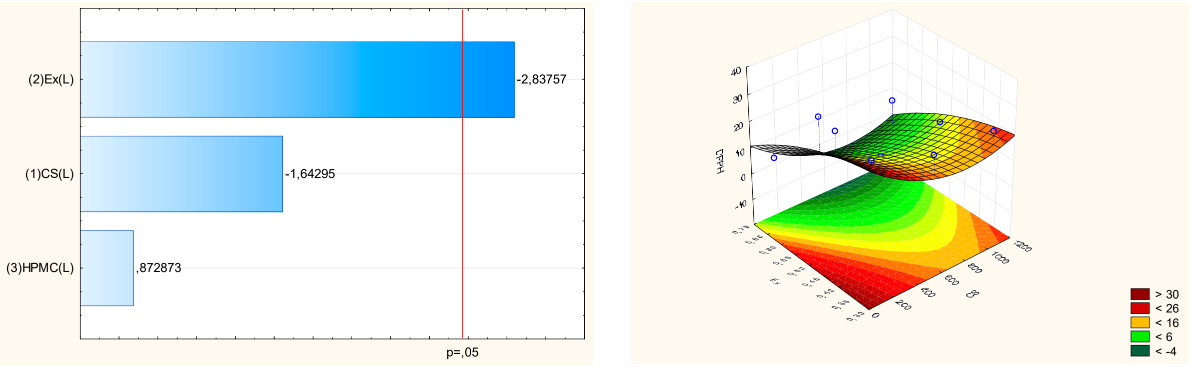

(a) (b)

Figure S6. Statistical analysis of antioxidant activity: (a) Pareto plot of standardized effects on antioxidant activity; (b) Response surface plots presenting the dependence of extract content and CS MW on the formulations' antioxidant activity for constant HPMC content of 12.5 %.

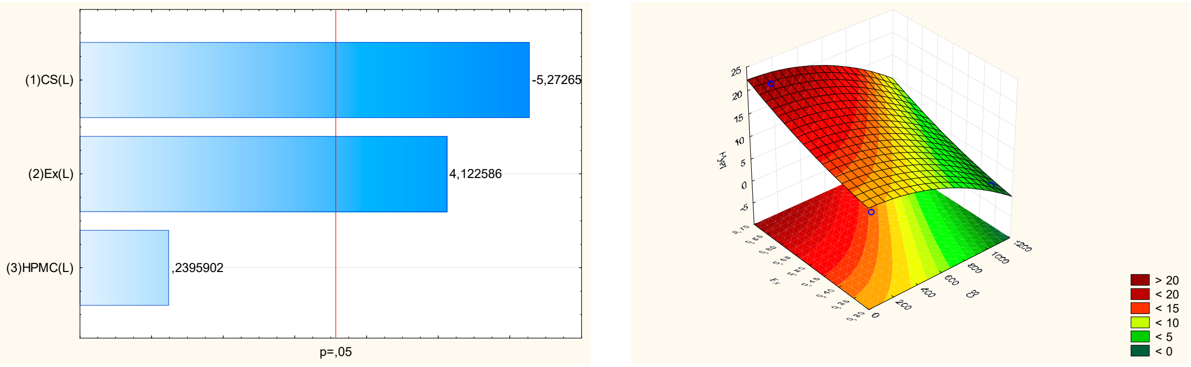

(a) (b)

Figure S7. Statistical analysis of anti-inflammatory activity: (a) Pareto plot of standardized effects on anti-inflammatory activity; (b) Response surface plots presenting the dependence of CS MW and extract content on the formulations' anti-inflammatory activity for constant HPMC content of 12.5 %.

Table S5. Correlation matrix.

	True density	Porosity	Dissolution	SI	DPPH	Hyal	Muco
True density	1.0000	-0.6859	**0.7744**	-0.5313	**-0.7664**	0.4873	-0.1432
Porosity	-0.6859	1.0000	**-0.8600**	**0.7313**	0.6491	-0.4470	0.2270
Dissolution	**0.7744**	**-0.8600**	1.0000	-0.6062	**-0.7788**	0.4563	-0.5161
SI	-0.5313	**0.7313**	-0.6062	1.0000	0.6161	-0.5137	-0.2966
DPPH	**-0.7664**	0.6491	**-0.7788**	0.6161	1.0000	-0.2433	0.1084
Hyal	0.4873	-0.4470	0.4563	-0.5137	-0.2433	1.0000	0.0782
Muco	-0.1432	0.2270	-0.5161	-0.2966	0.1084	0.0782	1.0000

Strong and very strong correlations are shown in bold.
